# [Corrigendum] Hsa_circularRNA_0079201 suppresses chondrocyte proliferation and endochondral ossification by regulating the microRNA-140-3p/SMAD2 signaling pathway in idiopathic short stature

**DOI:** 10.3892/ijmm.2026.5760

**Published:** 2026-02-09

**Authors:** Xijuan Liu, Chen Yan, Xueqiang Deng, Jingyu Jia

Int J Mol Med 46: 1993-2006, 2020; DOI: 10.3892/ijmm.2020.4737

Following the publication of the above article, an interested reader drew to the authors' attention that, concerning the Von Kossa staining experiments shown in Fig. 5E on p. 2002, the 'NC' and 'OvercircRNA-0079201+miR-140-3p mimic' data panels appeared to contain an overlapping section of data, such that data which were intended to show the results of different experiments had apparently been derived from the same original source. In addition, it was also noted that the COL10A1 western blots featured in Fig. 5D were strikingly similar to blots that had appeared in an article in *Journal of Cellular and Molecular Medicine* by the same research group.

In their response, the authors confirmed that the only figure part requiring correction was the 'NC' von Kossa staining panel in Fig. 5E; concerning the COL10A1 western blot in Fig. 5D, after re-examining the original experimental records and source files, they could confirm that this panel was derived from experiments conducted specifically for the above article.

The revised version of Fig. 5, now showing the correct data for the 'NC' data panel in Fig. 5E, is shown on the next page. The authors can confirm that the errors associated with this figure did not have any significant impact on either the results or the conclusions reported in this study, and all the authors agree with the publication of this Corrigendum. The authors are grateful to the Editor of *International Journal of Molecular Medicine* for allowing them the opportunity to publish this Corrigendum; furthermore, they apologize to the readership of the Journal for any inconvenience caused.

## Figures and Tables

**Figure 5:**
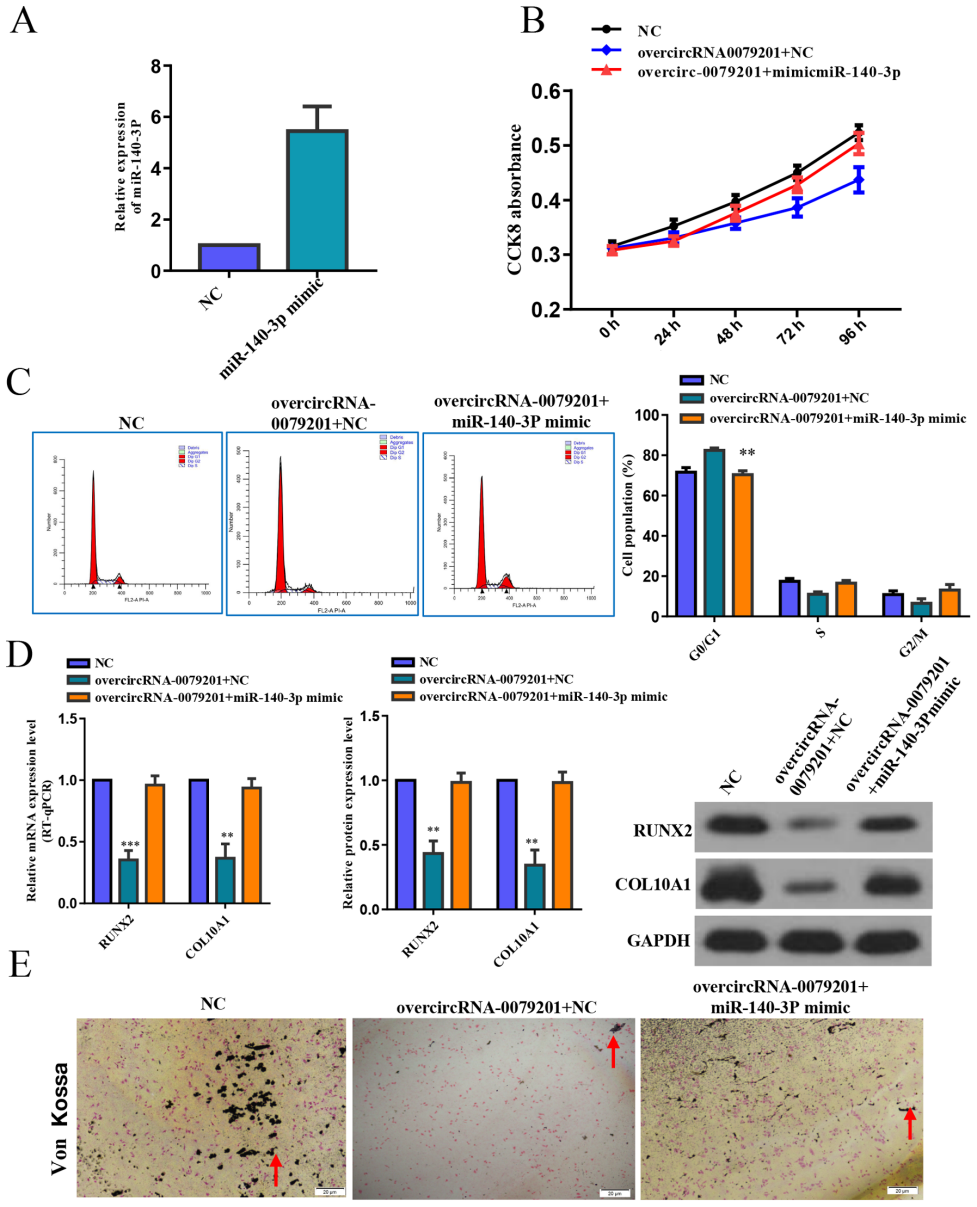
Rescue experiments indicates that miR-140-3p overexpression reversed the inhibition of human chondrocyte proliferation caused by overcir-cRNA_0079201. (A) The transfection efficiency of miR-140-3p mimics. (B) CCK-8 assay showed that overexpression of miR-140-3p reversed the human chondrocyte decrease in proliferation from the overcircRNA_0079201. (C) There was no significant difference in the number of cells in the different cell cycle phases between cells overcircRNA_0079201 +miR-140-3p and the NC, using flow cytometry. Rescue experiments showed that miR-140-3p overexpression reversed the inhibition of human chondrocyte (D) proliferation and hypertrophy and (E) endochondral ossification caused by overcircRNA_0079201. The data are presented as the mean ± SD. n=3. ^**^P<0.01 vs. control. CCK-8, Cell Counting Kit-8; NC, negative control; circRNA, circular RNA; miR, microRNA; COL10A1, collagen type X; RUNX2, runt-related transcription factor 2.

